# Resolution of vanishing bile duct syndrome in a patient associated with refractory hodgkin lymphoma following Anti–PD-1 therapy: a case report and literature review

**DOI:** 10.1007/s00277-026-06745-3

**Published:** 2026-02-26

**Authors:** Yi-Ching Lin, Wan-Chen Hsieh, Yu-Hsuan Tuan, Hsu-Hua Tseng, Jia-Huei Tsai, Tung-Hung Su, Tai-Chung Huang

**Affiliations:** 1 Department of Internal Medicine, Department of Internal Medicine, National Taiwan University Hospital, Taipei, Taiwan; 2 Division of Hematology and Medical Oncology, Department of Internal Medicine, Wan Fang Hospital, Taipei Medical University, Taipei, Taiwan; 3 Division of Hematology, Department of Internal Medicine, National Taiwan University Hospital, Taipei, Taiwan; 4 Division of Gastroenterology, Department of Internal Medicine, National Taiwan University Hospital, Taipei, Taiwan; 5 Department of Pathology, National Taiwan University Hospital, Taipei, Taiwan

**Keywords:** Vanishing bile duct syndrome, Hodgkin lymphoma, Anti–PD-1, Pembrolizumab, Cholestasis

## Abstract

Vanishing bile duct syndrome (VBDS) is a rare, and often fatal complication of Hodgkin lymphoma (HL), characterized by progressive intrahepatic bile duct loss and severe cholestasis. Management remains ill-defined, particularly in patients with refractory HL and significant hepatic dysfunction. We present a case of a young woman with biopsy-proven VBDS who experienced disease progression and worsening cholestasis despite second-line chemotherapy, corticosteroids, and brentuximab vedotin. Salvage therapy with pembrolizumab was initiated, resulting in a complete metabolic remission of HL and normalization of liver function. Notably, the patient did not experience immune-related hepatic adverse events. To our knowledge, this is the first report of HL-related VBDS successfully treated with programmed death-1 blockade. This case suggests that immune checkpoint inhibitors may be a viable therapeutic option for patients with HL-related VBDS, even in the setting of severe hepatic dysfunction.

## Introduction

Jaundice occurs in approximately 3–13% of patients with Hodgkin lymphoma (HL) and may arise from diverse etiologies, including hepatic infiltration, biliary obstruction, viral reactivation, drug-induced liver injury, hemolysis, or paraneoplastic syndromes [1, 2]. Vanishing bile duct syndrome (VBDS), a rare paraneoplastic manifestation characterized by progressive intrahepatic ductopenia and severe cholestasis, carries a particularly poor prognosis [3]. Currently, there is no established standard of care for HL-related VBDS [4].

Pembrolizumab, a programmed death-1 (PD-1) immune checkpoint inhibitor (ICI), has demonstrated robust efficacy in relapsed or refractory classical HL, particularly following brentuximab vedotin (BV) failure [5, 6]. To our knowledge, we report the first case of HL-related VBDS successfully treated with anti-PD-1 therapy.

## Case

A 23-year-old woman with no significant past medical history was diagnosed with Lugano stage IV nodular sclerosis classical Hodgkin lymphoma. She received induction chemotherapy with ABVD (doxorubicin, bleomycin, vinblastine, and dacarbazine), but subsequent positron emission tomography-computed tomography (PET-CT) demonstrated progressive disease. Salvage chemotherapy with DHAX (dexamethasone, high-dose cytarabine, and oxaliplatin) was initiated as a bridge to autologous stem cell transplantation.

Two weeks after initiating DHAX, the patient developed jaundice, tea-colored urine, and generalized pruritus. She denied fever, abdominal pain, arthralgias, myalgias, or acholia. Laboratory evaluation revealed a cholestatic pattern of liver injury: total bilirubin of 5.6 mg/dL, direct bilirubin of 3.5 mg/dL, aspartate aminotransferase (AST) of 119 U/L, alanine transaminase (ALT) of 278 U/L, and alkaline phosphatase (ALP) of 461 U/L. Workup for viral hepatitis, autoimmune liver disease, and metabolic etiologies was unremarkable. She reported no recent travel, animal exposure, alcohol consumption, or use of herbal supplements. Concomitant medications included prophylactic dose of cotrimoxazole (sulfamethoxazole 400 mg/trimethoprim 80 mg) and acyclovir 200 mg daily.

**Fig. 1:**
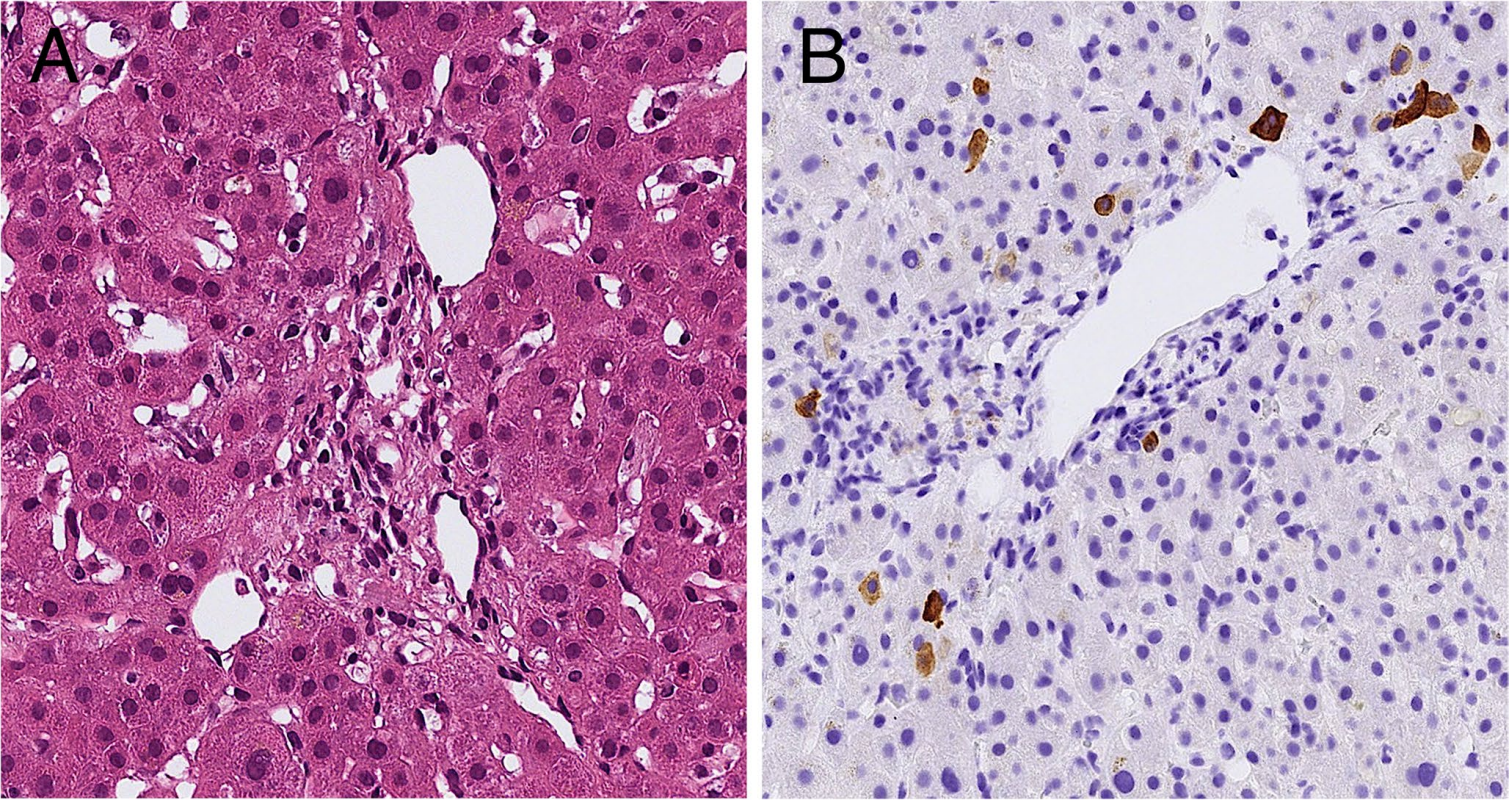
Liver biopsy findings. (**a**) Portal tract without bile duct (H&E, 29.48x). (**b**) Immunohistochemistry for cytokeratin 7 (CK7) shows absence of small bile ducts (CK7, 20.54×)

Abdominal computed tomography (CT) scan showed mild periportal edema and minimal ascites, with no evidence of hepatosplenomegaly or lymphadenopathy. Endoscopic retrograde cholangiopancreatography showed normal intrahepatic and extrahepatic biliary anatomy, without evidence of obstruction, stricture, or choledocholithiasis. A liver biopsy demonstrated cholestatic injury with extensive loss of small intralobular bile ducts in the majority of portal tracts, consistent with VBDS (Fig. 1a, b).

Therapy with prednisolone (30 mg daily) and ursodeoxycholic acid (600 mg daily) was initiated. This was followed by a prolonged tapering of dexamethasone for five months. Cotrimoxazole was discontinued approximately five months after the initial presentation. Despite these regimen, clinical and biochemical cholestasis persisted. A repeat liver biopsy performed after the corticosteroid course confirmed persistent cholestasis and ductopenia. Crucially, no Reed-Sternberg cells or atypical lymphoid infiltrates were identified. Although mild histologic improvement in ductopenia was noted compared to the initial biopsy, liver function remained severely impaired.

**Fig. 2:**
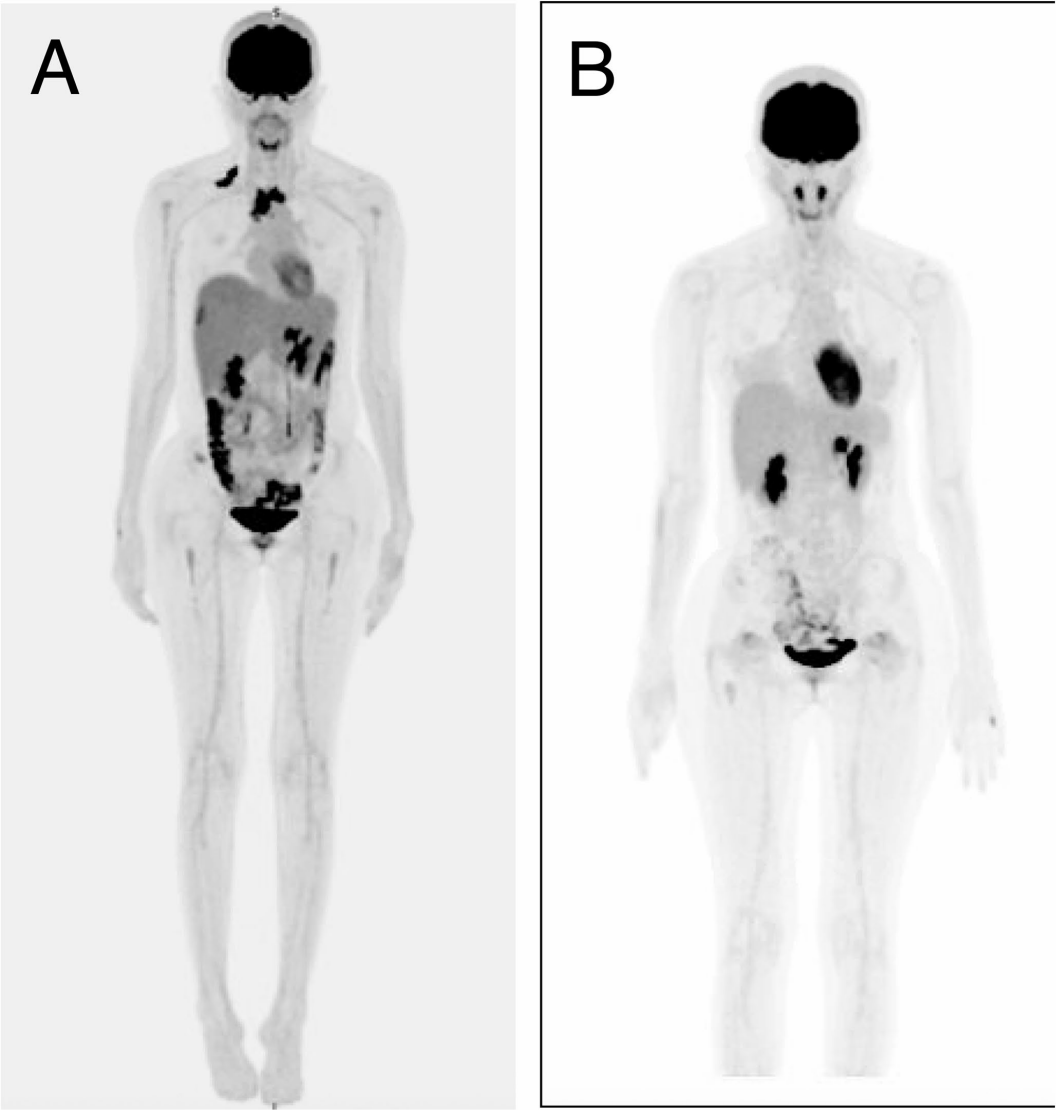
Positron emission tomography–computed tomography (PET-CT) scans. (**a**) PET-CT before brentuximab vedotin treatment shows active disease. (**b**) PET-CT after pembrolizumab therapy shows complete metabolic response

Follow-up PET-CT demonstrated disease progression (Fig. 2a). The patient was transitioned to BV as salvage therapy, initiated at a reduced dose of 1.2 mg/kg due to hepatic dysfunction. Following the first cycle, her serum bilirubin level acutely increased to 23.7 mg/dL, then gradually decreased to 15.1 mg/dL. Subsequent cycles were administered at the dose of 1.5 mg/kg, but hyperbilirubinemia persisted. In view of stable disease on interim imaging, gemcitabine and vinorelbine (GEV) were added to augment cytoreduction. The BV-GEV regimen comprised BV (1.8 mg/kg, day 1), gemcitabine (800 mg/m², day 1, 4), vinorelbine (5 mg/m², day 1), and oral prednisolone (100 mg daily, day 1–4). During the second cycle of this combination, the patient developed new-onset abdominal pain with worsening cholestatic markers (ALP and GGT). BV was discontinued due to suboptimal therapeutic response and exacerbation of hepatic cholestasis.

**Fig. 3:**
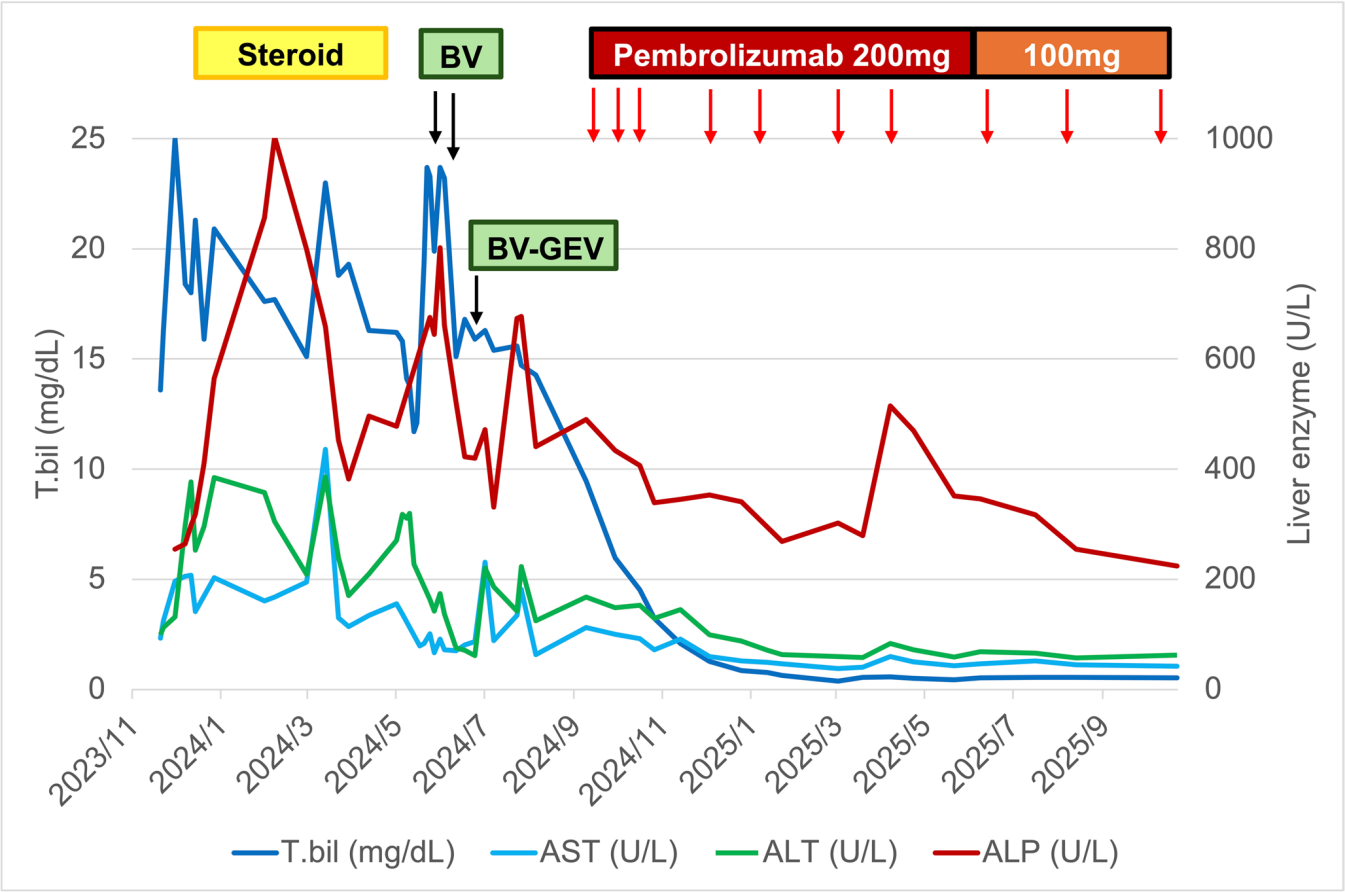
Liver function test results during treatment course. Treatments include corticosteroid monotherapy (Steroid), brentuximab vedotin (BV), BV combined with gemcitabine and vinorelbine (BV-GEV), and Pembrolizumab. AST, aspartate aminotransferase; ALT, alanine aminotransferase; ALP, alkaline phosphatase; T.bil, total bilirubin

Immunotherapy with pembrolizumab (200 mg every three weeks) was subsequently initiated. In the following cycles, serum bilirubin levels dramatically declined and normalized after four cycles of pembrolizumab (Fig. 3). Clinically, the patient’s symptoms resolved. Restaging PET-CT after seven doses of pembrolizumab demonstrated a complete metabolic response (Fig. 2b). The intervals of pembrolizumab were lengthened gradually, and she remains in the complete metabolic remission with close clinical surveillance.

## Discussion

This case demonstrates that in a patient with primary refractory classical HL complicated by VBDS, pembrolizumab was effective not only in controlling the underlying lymphoma but also in reversing the paraneoplastic hepatic dysfunction.

VBDS is a rare acquired disorder characterized by progressive destruction of intrahepatic bile ducts, leading to ductopenia. The association with HL was first described by Hubscher et al. in 1993 [3]. Since then, numerous cases have been reported, as summarized in Table 1. Although the precise pathogenesis remains unclear, proposed mechanisms include immune-mediated injury, paraneoplastic cytokine release, and T-cell cross-reactivity against biliary epithelial antigens [7–10].

**Table 1:** Reported literature involving the association between vanishing bile duct syndrome and hodgkin’s lymphoma

Author/Year	Pathology	HL treatment	Outcome/cause of death
Bouroncle/1962 [2]	IC	CT	Death/Hepatic failure
	IC	CT	Death/Hepatic failure
Juniper/1963 [25]	IC	Steroid alone	Death/Hepatic failure
Groth/1972 [26]	IC	CT	Death/Hepatic failure
Perera/1974 [27]	IC	RT + steroid	Remission
	IC	RT + steroid	Remission
	IC	RT + steroid	Death/Hepatic failure
Piken/1979 [28]	IC	CT	Death/Unknown
Trewby/1979 [29]	IC	CT	Remission
	Lymphoma infiltration	No treatment	Death/Hepatic failure
	Lymphoma infiltration	CT	Death/Unknown
	mild portal hepatitis	No treatment	Death/Hepatic failure
	mixed inflammatory and atypical histocytes	CT	Remission
	IC	CT	Death/Sepsis
Lieberman/1986 [30]	IC	No treatment	Death/Respiratory arrest
Birrer/1993 [31]	IC	CT	Death/Sepsis
Hubscher/1993 [3]	VBDS	CT	Death/Hepatic failure
	VBDS	CT + RT	Death/Hepatic failure
	VBDS	CT	Death/Hepatic failure
Jansen/1994 [32]	IC	RT	Death/Hepatic failure
Warner/1994 [33]	IC	*Yes	Remission
Gottrand/1997 [34]	VBDS	No treatment	Death/Hepatic failure
Crosbie/1997 [14]	VBDS	CT	Remission
De Medeiros/1998 [35]	VBDS	CT	Remission
	VBDS	CT	Death/Hepatic failure
Yalcin/1999 [36]	IC	No treatment	Sepsis
	IC	CT + RT	Remission
Dourakis/1999 [37]	Hepatocellular necrosis	CT	Death/Hepatic failure
Yusuf/2000 [38]	VBDS	No treatment	Death/Hepatic failure
Rossini/2000 [39]	VBDS	CT	Death/Hepatic
Allory/2000 [40]	VBDS	*Yes	Remission
Ozkan/2001[41]	VBDS	No treatment	Death/Hepatic failure
Ripoll/2002 [42]	VBDS	CT + RT	Death/Hepatic failure
	VBDS	CT + RT	Remission
Komurcu/2002 [43]	VBDS	CT, PBSCT	Death/Hepatic failure
Liangpunsakul/2002 [44]	Cholestatic hepatitis	CT	Remission
Guliter/2004 [45]	VBDS	CT	Death/Sepsis
Córdoba/2005 [46]	VBDS	CT	Remission
Han/2005 [47]	VBDS	No treatment	Spontaneous resolution of VBDS, Tumor recurrence
Schmitt/2006 [48]	VBDS	No treatment	Death/Sepsis
Barta/2006 [49]	IC	RT	Remission
DeBenedet/2008 [50]	VBDS	CT	Death/unknown
Leeuwenburgh/2008 [51]	VBDS	CT	Remission
Ballonoff/2008 [52]	VBDS	CT + RT	Remission
Pass/2008 [53]	VBDS	CT + RT	Death/unknown
	VBDS	CT + RT	progressive VBDS; HL remission
Umit/2009 [54]	VBDS	Steroid alone	Unknown
Wong/2013 [4]	VBDS	CT, PBSCT	Remission
Aleem/2013 [55]	VBDS	CT	Death/Hepatic failure
Nader/2013[56]	VBDS	CT	Death/Hepatic failure and Sepsis
Rota/2014 [57]	VBDS	CT	Remission
Bakhit/2017[58]	VBDS	CT + RT	Remission
Gupta/2016 [16]	VBDS	BV + CT	Remission
	VBDS	BV + CT	Remission
Fong/2019 [17]	VBDS	BV + CT	Remission
Papakonstantinou/2021[18]	IC	BV + CT	Remission
Ishitsuka/2022 [19]	VBDS	BV	Remission
Morgan/2023 [20]	VBDS	BV + CT	Remission
Ma/2025 [21]	VBDS	BV + CT	Remission
Our case	VBDS	Pembrolizumab	Remission

*, no reported regimen; BV, brentuximab vedotin; CT, chemotherapy; HL, Hodgkin lymphoma; IC, intrahepatic cholestasis; PBSCT, peripheral blood stem cell transplantation; RT, radiotherapy; VBDS, vanishing bile duct syndrome

Accurate liver assessment is critical in Hodgkin lymphoma. Picardi et al. [11, 12]established Color Doppler ultrasound-guided core-needle biopsy (CNB) as a safe, high-yield technique for distinguishing lymphoma infiltration from infection or toxicity, even in patients with severe coagulopathy. Beyond diagnosis, ultrasound is effective for surveillance; a randomized trial [13] confirmed its utility in detecting relapse during first remission. Consequently, ultrasonography may be integrated into management algorithms to ensure both safe histological characterization and effective, radiation-free monitoring.

While HL-related VBDS was potentially reversible in some case reports, the overall prognosis remains poor, primarily due to hepatic failure or sepsis (Table 1). The management of HL- related VBDS remains challenging. Conventional modalities, including chemotherapy, radiotherapy, corticosteroids, and supportive care, have demonstrated limited efficacy. While some experts advocate dose-attenuated regimens in patients with hepatic dysfunction, others support full-dose chemotherapy when clinically feasible [4, 11].

BV, an anti-CD30 antibody drug conjugate, has demonstrated efficacy in relapsed HL [15]. However, its application in patients with severe hepatic impairment remains controversial. Several case reports suggest that BV monotherapy or BV combined with chemotherapy may help transition HL patients with severe liver dysfunction to partial hepatic recovery for potential subsequent intensive treatment. Nevertheless, BV alone often does not achieve complete response, particularly in refractory cases [16–21].

In contrast, ICIs such as pembrolizumab and nivolumab have established robust efficacy in relapsed or refractory HL. In our patient, pembrolizumab was initiated after the failure of both conventional chemotherapy and BV. This resulted in the complete resolution of both cholestasis and the lymphoma, without precipitating hepatic immune-related adverse events (irAEs). Although hepatotoxicity has been reported in up to 6% of patients receiving PD-1 inhibitors, it typically manifests as transaminase elevation rather than cholestasis. Paradoxically, VBDS itself has been described as a rare immune-mediated complication of pembrolizumab [22–24]. However, in this case, VBDS was attributable to HL as a paraneoplastic syndrome, and pembrolizumab achieved a complete metabolic response and resolution of cholestasis by controlling the underlying malignancy.

This apparent paradox underscores the heterogeneity of VBDS pathogenesis. The syndrome may arise from diverse mechanisms, and optimal management must therefore be individualized according to the underlying etiology. This mechanistic variability may explain why pembrolizumab can precipitate VBDS as an irAE in some cases, yet provide therapeutic resolution in HL-associated VBDS. Clinicians should carefully weigh the clinical context when selecting therapy and remain vigilant for immune-mediated sequelae. Nonetheless, our case suggests that PD-1 inhibition represents a promising and well-tolerated salvage option for selected patients with HL-related VBDS, particularly who are not eligible for conventional therapy.

## Conclusion

HL-related VBDS is a rare, life-threatening complication that presents significant therapeutic challenge. Our case highlights the potential role in pembrolizumab to bring refractory HL into remission even in the context of VBDS, without inducing hepatic irAEs. Our report captures this importance. Further studies are warranted to validate the safety and efficacy of immune checkpoint inhibitors in this type of clinical conundrum.

## Data Availability

The datasets generated during and analyzed during the current study are available from the corresponding author upon reasonable request.
